# The effect of missing data on linkage disequilibrium mapping and haplotype association analysis in the GAW14 simulated datasets

**DOI:** 10.1186/1471-2156-6-S1-S151

**Published:** 2005-12-30

**Authors:** Pamela A McCaskie, Kim W Carter, Simon R McCaskie, Lyle J Palmer

**Affiliations:** 1 Laboratory for Genetic Epidemiology, Western Australian Institute for Medical Research, UWA Centre for Medical Research, University of Western Australia, Ground Floor, B Block, Hospital Avenue, Nedlands, Western Australia

## Abstract

We used our newly developed linkage disequilibrium (LD) plotting software, JLIN, to plot linkage disequilibrium between pairs of single-nucleotide polymorphisms (SNPs) for three chromosomes of the Genetic Analysis Workshop 14 Aipotu simulated population to assess the effect of missing data on LD calculations. Our haplotype analysis program, SIMHAP, was used to assess the effect of missing data on haplotype-phenotype association. Genotype data was removed at random, at levels of 1%, 5%, and 10%, and the LD calculations and haplotype association results for these levels of missingness were compared to those for the complete dataset. It was concluded that ignoring individuals with missing data substantially affects the number of regions of LD detected which, in turn, could affect tagging SNPs chosen to generate haplotypes.

## Background

As we begin to discover more about how haplotypes are defined and inherited, the emphasis in genetic association studies has moved away from the analysis of single nucleotide polymorphisms (SNPs) to incorporate multilocus haplotype analysis. Individuals often inherit a set of syntenic SNPs in linkage disequilibrium (LD) from one parent as a unit commonly termed a haplotype. LD, which refers to the non-independence of alleles in haplotypes, provides us with information about the statistical non-independence of markers. Haplotypes are likely to play a key role in helping us to understand the genetic basis of complex human diseases. In principle, haplotypes should offer advantages in terms of statistical power to detect a true association with a given sample size compared with analyses based on single SNPs or combinations of SNPs [1-3], because they contain more genetic information than the genotypes alone.

It is increasingly clear from other fields of statistical investigation that simply ignoring missing data or restricting the analysis to subjects with complete data-even when data is missing completely at random-can lead to biased or inefficient analyses [4-8]. This problem worsens if data are not missing at random, as may be the case with systematic errors in genotyping assays, and hence imputation of such data can be difficult without information about the reason for the missingness. Ignoring individuals with missing data is an inadequate way of dealing with the problem, although it is often the procedure adopted. However, in the case of missing genetic data, the effects on LD analysis and subsequent haplotype formation can be substantial depending on the amount of data missing.

Another known problem with association analyses using haplotypes is the uncertainty around inferred haplotypes when phase is ambiguous for an individual. For individuals with multiple heterozygous loci, more than one haplotype pair (or diplotype) is possible. The most commonly used method for haplotype implementation is to determine the most likely diplotype for an individual with ambiguous phase, and treat it as known. This does not however, take into account the possibility that the most likely haplotype is not correct. Information surrounding the likelihood of an allocated diplotype can be used in association testing to weight diplotypes given their probabilities of being true.

It was the aim of this contribution to use the Genetic Analysis Workshop 14 (GAW14) simulated data to assess the effects of varying degrees of missing data on LD and on haplotype association with Kofendrerd Personality Disorder (KPD), using JLIN [9] and SIMHAP [10]. We hypothesized that increasing proportions of missing data would result in a decreased ability to detect regions of LD and a concomitant reduction in power to detect haplotype associations.

## Methods

### Generating missing data

The GAW14 simulated data provided a useful platform for testing the effects of missing data on the calculations of LD coefficients and haplotype-phenotype associations across a region. As this data was complete, we were able to calculate LD for the set of markers on a chromosome and treat this as a baseline to which we compared the LD results when varying degrees of missing data were generated. We were blind to the answers at the time of analysis. Using replicate 1 of the simulated data from the Aipotu region, genotypes were removed at random from chromosomes 1, 3, and 10. Explanation for choosing these chromosomes is provided in the Haplotyping section below. New datasets were generated with genotype missingness implemented at rates of 1%, 5%, and 10%. Data were removed over all markers rather than at individual markers. The initial 1% of genetic data removed was included in the 5% missing dataset, and this 5% of genotypes removed were then included in the 10% of data to be removed for the final set, to ensure consistency.

### Mapping LD

JLIN is an LD visualization program that we have developed that derives various disequilibrium coefficients for pairs of biallelic markers on a chromosome. Two-SNP haplotype frequencies are derived using the expectation-maximization (EM) algorithm for phase uncertainty. The disequilibrium coefficient D' as described by Lewontin [11] is calculated, and various other disequilibrium coefficients are derived as described by Devlin and Risch [12]. LD information is displayed in a graph, where the user can easily specify parameters such as axes labels, colors, and statistics displayed. D' was plotted for the three generated datasets, as well as for the original complete dataset on chromosomes 1, 3, and 10 within the four simulated populations. These plots were compared to assess if any obvious differences were apparent across the varying levels of missing data.

### Haplotyping

As part of another contribution to GAW14, we performed linkage and association analyses using MERLIN [13] and QTDT [14], respectively. From these analyses several regions of markers showed significant linkage and association with disease status, in particular, regions surrounding C01R0050, C03R0280, and C10R0882. Using LD visualization and the program BEST [15], we defined a set of tagging SNPs to define haplotype blocks in these regions for the Aipotu population. The set of tagging SNPs surrounding C01R0050, C03R0280, and C10R0882 were termed region 1, region 2, and region 3, respectively. New datasets were generated where genotype missingness was implemented at rates of 1%, 5%, and 10% within the tagging SNPs, using the same method described in the previous section.

SIMHAP is a program that we have developed for haplotype association analysis. It uses an EM algorithm as described by Excoffier and Slatkin [16] to impute diplotypes in individuals, determining a posterior probability for each possible diplotype for an individual. It then utilizes this information to simulate through generated datasets, where the diplotype for individual *i *in a given dataset is sampled from *n *possible diplotypes for that individual with the given posterior probability. This ensures that the possibility that the most probable diplotype is not the actual diplotype is addressed and information surrounding less probable (but not impossible) diplotypes is incorporated. As a result, an empirical distribution of parameter estimates, taking into account uncertainty around haplotype inference, can be derived. The mean, as well as the 95% confidence interval of the mean, for each parameter estimate over the empirical distribution is returned. SIMHAP currently does not infer haplotypes for pedigree data and so diplotypes with their posterior probabilities were derived using the program HAPLO [8], which calculates these values for individuals within pedigrees, and imported into SIMHAP for use in the modelling process.

The original, complete data and the datasets generated with missing data were run through SIMHAP to model the effect of haplotypes defined by our tagging SNPs on affection status. Results were derived from 10,000 simulations of the model to ensure that diplotypes with small posterior probabilities were included in the sampling process. For each simulation, a generalized linear mixed model was performed, with family ID as a clustering variable. The coefficients and their respective significance values for haplotypes of interest were extracted from each fitted model to form an empirical distribution, and the means and 95% confidence intervals of these estimates were returned.

## Results

### LD estimation

**Figure 1:**
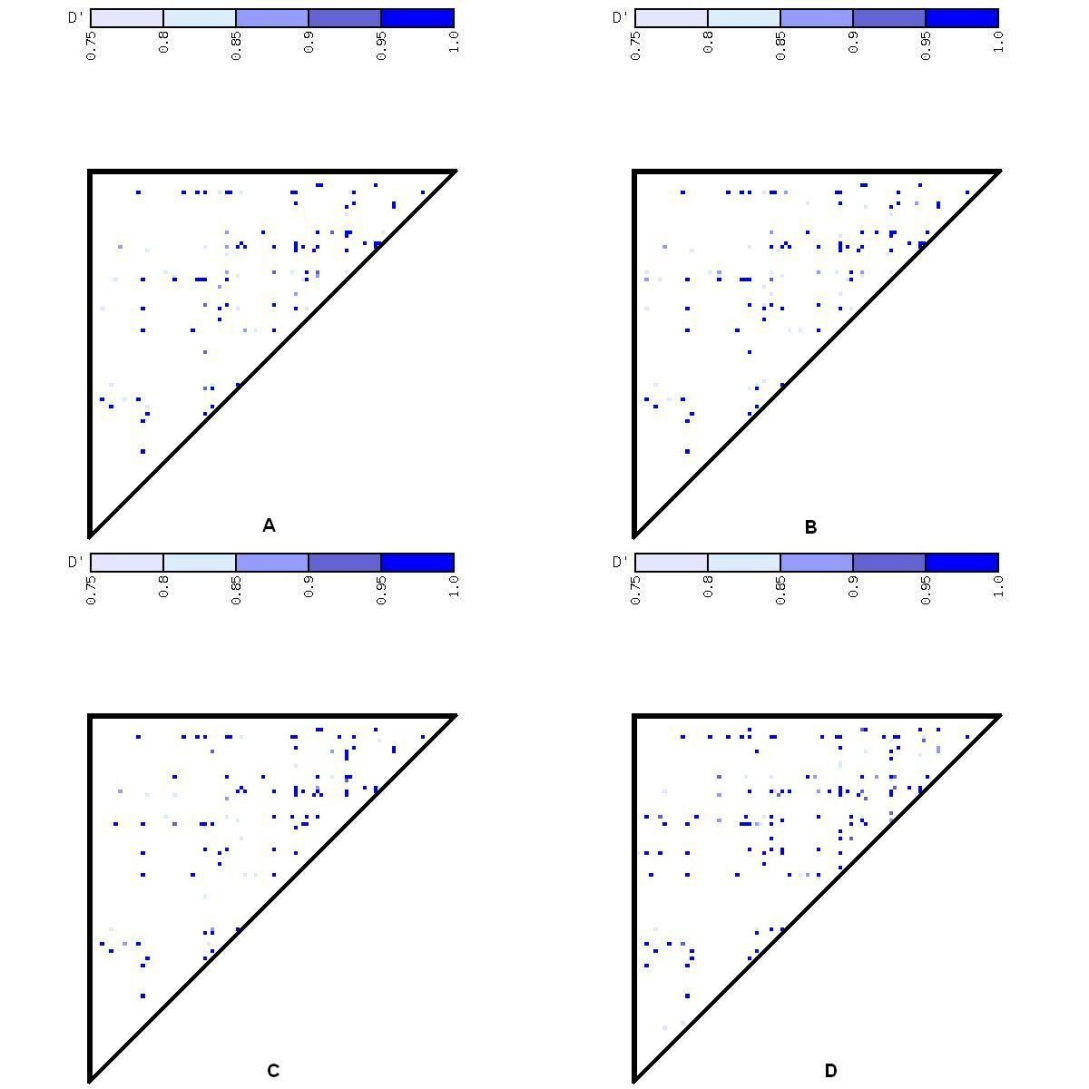
**Plots of the LD coefficient D' for region 2 in the Aipotu population**. A, Full dataset with no missing values; B, 1% missing genotypic data; C, 5% missing genotypic data; D, 10% missing genotypic data.

**Figure 2:**
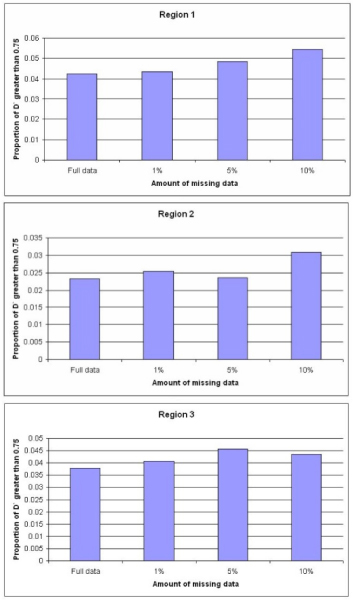
**Varying proportions of strong D' with increasing degrees of missing data**. Histograms of the proportion of pair-wise comparisons exhibiting a D' value greater than 0.75 for varying degrees of missing data.

Figure 1 shows plots of the LD coefficient D' for pairs of markers in region 2 for the Aipotu population. An incremental shading scale is used to display D' values, where darker shading indicates a higher D' value and thus stronger LD. Only D' values greater than 0.75 are plotted because this is a widely accepted indicator of strong LD. Plot A represents the complete dataset with no missingness. Plots B, C, and D are LD maps for data missing at a rate of 1%, 5%, and 10%, respectively. The LD maps in B, C, and D show increasing amounts of high LD when compared with Plot A. The most evident difference can be observed when comparing plots A and D, where regions of strong LD (indicated by a high D') appear to be more frequent in plot D than in plot A. The plots for the remaining two regions studied exhibit similar effects to those shown for region 2, suggesting that the effect is not population- or chromosome-specific. Because this effect is difficult to appreciate from the LD plots alone, the proportion of pair-wise SNP comparisons exhibiting a D' greater than 0.75 for each level of missing data was determined. Figure 2 shows histograms of these proportions for the three regions studied and it can be seen that generally, these proportions increase as missing data increases.

### Haplotype analysis

Five haplotype tagging SNPs were defined to characterize a haplotype block in region 2. Two haplotypes in this region were found to be associated with (protective for) KPD. These SNPs were: B03T3041, B03T3046, B03T3050, B03T3058, and B03T3064. H12121 and H12221 were the two associated haplotypes. H12121 represents a haplotype composed of allele 1 of marker B03T3041, allele 2 of marker B03T3046, allele 1 of marker B03T3050, allele 2 of marker B03T3058, and allele 1 of marker B03T3064. Haplotype H12221 is similarly defined. Table 1 presents the odds ratios, standard error of the log odds ratios, and *p*-values determined by SIMHAP for each haplotype over the varying degrees of missing data. There is a trend towards increased 95% confidence intervals for all measures as the amount of missingness increases, although no obvious patterns emerge relating to changes in haplotype effects. No haplotypes in region 1 or region 3 were found to be significantly associated with affection status for any degree of missing data.

## Discussion

The analysis of LD between markers is an important factor for LD mapping and association, and is important in determining haplotypes. The GAW14 simulated data provided a useful platform for assessing the effect of missing data at varying levels, on LD calculation and haplotype estimation. Using the LD plotting program JLIN, we discovered that simply ignoring missing genotype data affects how accurately we map regions of LD, and the level of strong LD observed. Our hypothesis of a decrease in detectable LD with increased missing data was not supported. The number of pair-wise comparisons exhibiting strong LD tended to increase as missing data increased. As this number increases, the pattern of LD across a chromosome can become more segregated, causing the partitioning of haplotype blocks into smaller blocks. This resulting loss of the overall pattern of LD could lead to problems in with tag SNP selection and haplotype formation.

The set of haplotype tagging SNPs for haplotype blocks was generated for the complete dataset and then missing data was generated within these genotyped SNPs. It is possible that the set of tagging SNPs may have been different if they had been chosen after the removal of data; however the aim of our analysis was to assess the effect of missing genotype data during haplotype analysis. In reality, missing data due to genotyping errors will occur after the selection of tagging SNPs (as these are the markers genotyped in a population of interest), and thus the effect of missing data on detection of haplotype association is of more practical concern. A trend toward increasing confidence intervals around parameter estimates did emerge as the amount of missing data increased, however the parameter estimates themselves remained relatively unchanged and the amount by which the intervals increased formed no distinct pattern. This suggests that haplotype association analysis is fairly robust to missing data. The increasing confidence intervals could simply be a reflection of decreased power due to a smaller dataset.

## Conclusion

The most common practice for dealing with missing data in genetic analysis is to simply remove or ignore individuals with missing data. We have shown, using plots of LD that this practice affects the LD coefficient D' and can result in an increase in number of pair-wise comparisons exhibiting strong LD. The large effect of missing data on LD did not directly translate into large effects on haplotype analysis. This suggests that haplotype formation and analysis is fairly robust to missing data up to a level of 10%. LD is a powerful tool in determining haplotype blocks and if strong areas of LD are wrongly defined when data missingness is large, this could affect the way that we determine haplotype tagging SNPs and thus affect haplotype formation. More research could help to determine the effect of missing data on haplotype tagging SNP selection. Future work is proposed for exploring various methods of data imputation, and techniques such as those adopted in this paper can be used to help determine how effectively different imputation methods perform.

## Abbreviations

EM: Expectation maximization

GAW14: Genetic Analysis Workshop 14

KPD: Kofendrerd personality disorder

LD: Linkage disequilibrium

SNP: Single-nucleotide polymorphism

## Authors' contributions

PAM performed the haplotype analyses, aided with the LD mapping, and drafted the manuscript. KWC generated the missing data for LD, created the LD plots, and drafted the manuscript. SRM provided programming support during the development of SIMHAP and the haplotype analyses. LJP conceived of the study, participated in the design and coordination of the study, and assisted in drafting the manuscript.

## Figures and Tables

**Table 1:** Haplotype results for varying degrees of missing data

	OR (95% CI)	SE (logOR) (95% CI)	*p*-Value (95% CI)
H12121			
Complete data	0.52 (0.49, 0.57)	-0.64 (-0.71, -0.57)	0.011 (0.005, 0.022)
1% Missing data	0.57 (0.53, 0.61)	-0.56 (-0.64, -0.49)	0.027 (0.012, 0.052)
5% Missing data	0.58 (0.53, 0.63)	-0.54 (-0.63, -0.46)	0.034 (0.013, 0.070)
10% Missing data	0.52 (0.46, 0.58)	-0.66 (-0.77, -0.54)	0.010 (0.002, 0.029)
H12221			
Complete data	0.62 (0.58, 0.65)	-0.48 (-0.54, -0.43)	0.021 (0.010, 0.037)
1% Missing data	0.61 (0.57, 0.64)	-0.50 (-0.57, -0.44)	0.018 (0.007, 0.034)
5% Missing data	0.59 (0.54, 0.64)	-0.52 (-0.61, -0.44)	0.014 (0.004, 0.034)
10% Missing data	0.59 (0.53, 0.65)	-0.53 (-0.63, -0.43)	0.014 (0.003, 0.039)
